# Application Effect of Poly-L-Lactic Acid Filler in Surgical Repair of Scar Contraction After Nasal Implant Surgery: Poly-L-Lactic Acid Filler in Surgical Repair of Scar Contraction

**DOI:** 10.31661/gmj.vi.3699

**Published:** 2025-05-27

**Authors:** Xiang Wang

**Affiliations:** ^1^ University and School BAB Medical Beauty Hospital, Sichuan Chengdu 610037, China

**Keywords:** Poly-L-lactic Acid, Nasal Implant Surgery, Scar Contraction, Repair, Application

## Abstract

**Background:**

Nose implant procedure is one of the most common aesthetic surgeries that can be performed, but patients frequently experience complications like scar contracture that’s quite bothersome. Numerous treatment approaches have been attempted, most notably with the Poly-L-lactic Acid Filler (PLLA) which seems to offer some promise. In this paper, we will attempt to assess the use of PLLA filler in surgically correcting scar contractures associated with nose implant surgery, this study aims to explore the application effect of Poly-L-lactic Acid Filler in surgical repair of scar contraction after nasal implant surgery and provide some reference value for relevant research.

**Case Presentation:**

nose implant procedure is one of the most common aesthetic surgeries that can be performed, but patients frequently experience complications like scar contracture that’s quite bothersome. Numerous treatment approaches have been attempted, most notably with the Poly-L-lactic Acid Filler (PLLA) which seems to offer some promise. In this paper, we will attempt to assess the use of PLLA filler in surgically correcting scar contractures associated with nose implant surgery.

**Conclusion:**

It is highly suggested that PLLA Filler is a valid treatment option for scar contracture repair enhancement after nasal implant procedures. However, it’s best practice to verify long-term results on larger sample sizes.

## Introduction

The nose, located in the center of the face, is a critical area that contributes to facial appearance; Therefore, it has received significant attention and importance from people [1]. In recent years, rhinoplasty (nasal implant surgery) has become one of the main surgical procedures in the field of cosmetic surgery. Research results have shown that some patients who undergo rhinoplasty may experience scar contracture on the nasal dorsum due to differences in their basic conditions or differences in the quality of the surgery [2]. For some eligible patients, scar release treatment can be performed for repair. This method mainly disrupts the scar tissue by blunt dissection and repairs it by filling with a needle injection, which stimulates new collagen regeneration and thus repairs the scar tissue [3]. However, in practical clinical work, it has been found that some patients with nasal scar contracture have a higher risk of embolism after injection of filler due to the uncertainty of blood vessel direction, resulting in serious adverse consequences and the failure to meet the needs of patients [4]. Therefore, the requirements for filling materials in nasal implant surgery are relatively high. Poly-l-lactide (PLLA) is a product made from poly-L-lactic acid, which has good biocompatibility and a very low risk of embolism and is commonly used as an organ support material in the medical field. Using PLLA as a material for contour filling and modification in the scar area may reduce the risk of embolism after surgery, reduce the occurrence of complications, and improve the repair effect of scar contracture. In view of this, based on the author’s clinical work in treating one scar contracture patient with PLLA and achieving good repair effects, this paper aims to provide some reference value for relevant research.

## Case presentation and treatment process

**Figure-1:**
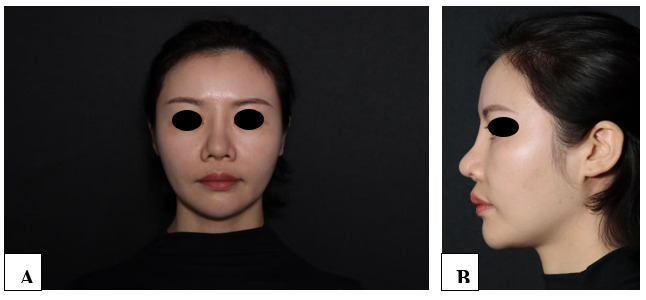
Figure 1. Frontal view (A) and profile view (B) before surgery

**Figure-2:**
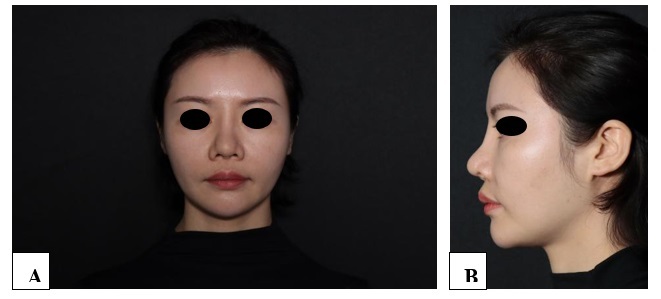
Figure 2. Immediate postoperative front view (A) and side view (B)

**Figure-3:**
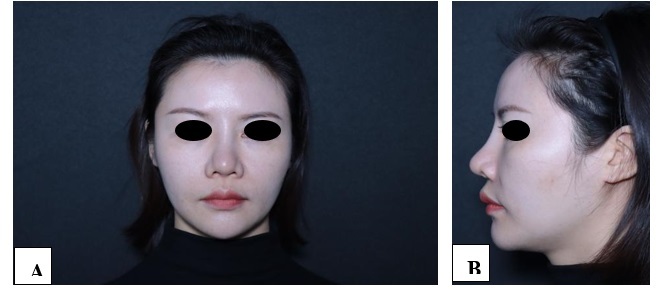
Figure 3. One-month postoperative front view (A), and profile view after the first surgery (B).

**Figure-4:**
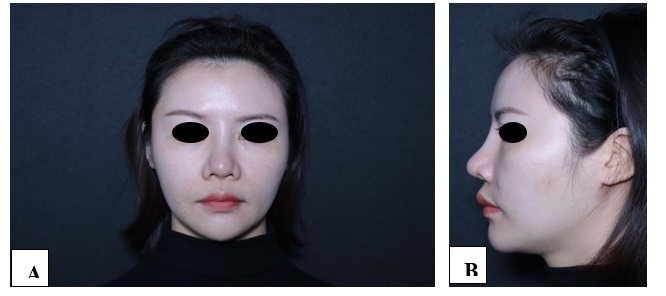
Figure 4. One-month postoperative front view (A) and profile view (A) after the second surgery.

**Figure-5:**
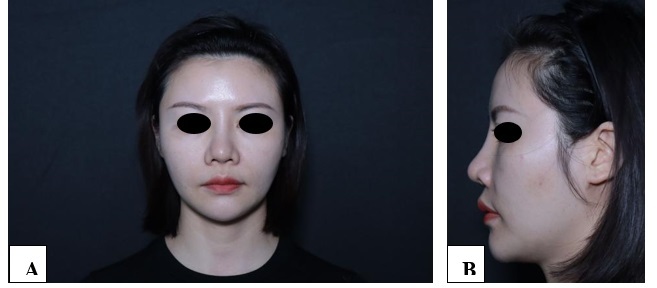
Figure 5. One-month postoperative front view (A) and profile view (B) after the third surgery.

**Figure-6:**
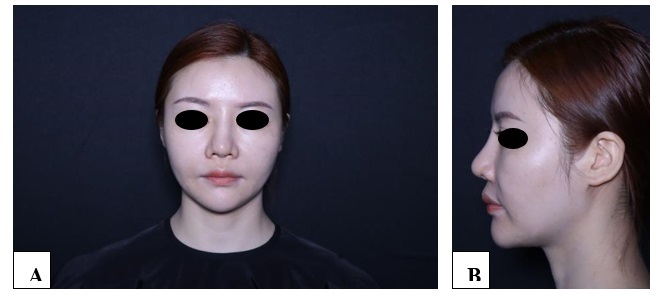
Figure 6. One-month postoperative front view (A) and profile view (B) after the fourth surgery.

### 1. Medical history

The patient, 32-year-old female, underwent nasal implant surgery in August 2022. Four months after the surgery, she experienced an obvious collapse of the nasal dorsum, which severely affected her appearance. She was admitted to our hospital in September 2022.

### 2. Admission Examination

The vital signs of the patient were stable, and no obvious abnormalities were found during the physical examination. The patient had a short and small nose, with visible scars on the nasal dorsum. After examination, the patient was diagnosed with "scar contracture" and proposed to undergo surgical treatment.

### 3. Treatment method

The patient underwent scar release surgery, in which the scar tissue was bluntly dissected, and filler was injected to repair the nasal dorsum. The treatment was performed once a month for a total of four times. The specific steps were as follows:

Under general anesthesia, an open-approach nasal plastic surgery incision was made. During the dissection of the columellar flap, the thickness of the columellar flap was kept at least 1.5 mm to avoid postoperative vascular disorders. If the outer lateral cartilage could be found, the dissection plane was located on the surface of the cartilage membrane. If it was difficult to find, enough thickness of skin and soft tissue should be preserved. The skin and soft tissue were pulled downward with a double hook to reveal the scar contracture band of the skin and soft tissue layer. The band was completely interrupted. The dome was split from the midline to reveal the lateral edge of the lower lateral cartilage. The lower lateral cartilage was pulled toward the tail to expose the contracture band in the scroll area, which was completely released. If necessary, the edge of the head was cut open to reveal the outer lateral foot. The scar on the surface of the cartilage was removed in the plane of the cartilage membrane, and the cartilage was fully stretched. The cartilage was separated in a retrograde manner towards the dome and inner lateral foot, and the scar was released with a blunt needle. Filler was injected to repair the nasal dorsum, with four treatments performed once a month.

### 4. Preparation of filling solution

The injection filling solution used in the treatment was Avilane brand poly-L-lactic acid reagent bottle (product specification: 340mg/bottle, diluted with 5 ml). For the area above the SMAS layer, 5 ml was used for dilution, and for the area below the SMAS layer, 3 ml was used for dilution. After dilution, it became a suspension, and the injection dose was 1 ml.

### 5. Results

With each successive procedure, the repair of the patient’s nasal dorsal scar showed progressively more pronounced improvements compared to the previous operation. By the fourth procedure, the patient exhibited substantial recovery, with complete healing achieved within one month. Throughout the treatment process, no adverse complications, including thrombosis, nodules, deformities, or inflammation, were observed. These results suggest a positive response to the interventions, indicating their effectiveness in promoting healing without significant post-operative issues. For further details, please refer to Figures 1 2 3 4 5 6 7.

### 6. Outcome Measures

#### 6.1. Standardized Photography

Standardized digital images, consisting of frontal, 45-degree oblique, and profile views were taken at the baseline visit prior to each intervention session and then at the final 6-month follow-up visit using VISIA-CR imaging system provided by Canfield Scientific, Inc., Fairfield NJ, USA. To make possible comparisons, equivalent lighting, posture, and camera settings were always used during all Figure sessions.

### 6.2. VISIA Skin Analysis

Quantitative skin analysis was performed using the VISIA system at baseline and the 6-month follow-up. The following parameters were assessed:1) Texture: A measure of skin smoothness and evenness.2) Pores: Quantification of pore size and visibility.3) Spots: Assessment of brown and red discolorations, including post-inflammatory hyperpigmentation.4) Wrinkles: Evaluation of fine lines and wrinkles.5) UV spots: Identification of sun damage and underlying pigmentation issues.

### 6.3. ECCA Grading

The severity of acne scars was graded using the Échelled›Évaluation Clinique des Cicatrices d’Acné (ECCA) grading scale [5]. This is a validated scale that takes into account both scar morphology and density. Higher scores on this scale reflect greater severity of scarring. Two independent dermatologists scored the patients at baseline and at 6 month follow-up using the ECCA scoring scale.

### 7. Assessment Results of Repair Effectiveness

This is quantitatively assessed through reductions in ECCA scores, which indicate a decrease in scar severity, and improvements in metrics such as skin texture, pore size, firmness, pigmentation, and luster, as evaluated by a comprehensive skin quality grading system. Additionally, qualitative indicators include high levels of patient satisfaction and minimal adverse events, reflecting both the aesthetic and functional benefits of the treatment. These outcomes collectively represent the "good effect on repairing" achieved with PLLA injections.

## Ethics Statement

This study follows the "Helsinki Declaration", this study has been approved by our hospital’s ethics committee (Ethics Approval No.: 2023-03-25). The images and medical records involved in the study have been fully informed and consented to by the case subjects, and the informed consent form has been signed.

## Discussion

**Figure-7:**
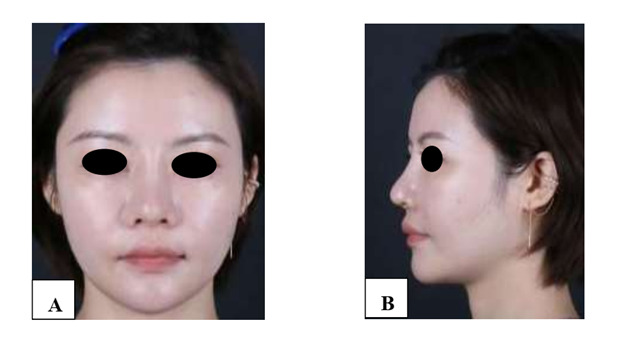
Figure 7. Six-month postoperative front view (A) and profile view (B).

In recent years, with the widespread popularity of rhinoplasty, the number of people undergoing nose augmentation has increased, and with it the risk of postoperative nasal scar contracture deformities. Nasal scar contracture is usually due to incomplete tissue repair after rhinoplasty, resulting in scar tissue. Surgical scar release is currently the mainstream method for repairing scar contracture on the nasal dorsum. However, because there is also widespread fibrotic scarring in the nasal dermis, the effect of extensive dissection and release is limited, and there is a risk of affecting skin blood flow. Therefore, many plastic surgeons have begun to try adjunctive treatment strategies, such as injecting autologous fat extract products to assist in tissue regeneration and blood vessel formation in the nasal region, for repair purposes. According to relevant literature, An et al. achieved significant improvement in the nasal parameters of patients by extracting micro-fat particles and performing three subcutaneous injections while manually pulling the skin, and using autologous rib cartilage for repair [6]. Oh et al. extracted fat-derived matrix cells by centrifugation, enzymatic digestion, and secondary centrifugation of autologous fat, and performed multiple subcutaneous injections at the nasal tip and areas of skin stiffness [7]. Ahn et al. used the autologous fat extract to extract fat-derived matrix vascular components (ADSVF) for pre- and post-operative multiple subcutaneous injections for repair [8]. These fat extracts have the characteristics of promoting tissue regeneration and inducing blood vessel formation, which can promote skin softening and expansion in the nasal region [9][10]. However, injecting fat extracts carries a certain risk of embolism, and related complications are also frequently reported. In addition, most clinical experience indicates that fat filling may cause pain and swelling, which may significantly reduce patient comfort in the days following surgery [11].

PLLA is a biocompatible, biodegradable, and absorbable polymer. It is an α-hydroxy acid polymer of L-lactic acid and has been safely used in the medical field for over 30 years as absorbable sutures and as implants for bone and soft tissue [12]. Currently, injectable PLLA has been successfully used for correcting nasolabial folds, sagging chin, and other facial aging treatments. It was first introduced as a "facial filler" for HIV patients in 2004. Studies have shown that PLLA can significantly stimulate collagen production and its effects are long-lasting [13]. Therefore, it is widely used for treating facial volumes, contouring, skin laxities, fat pads, scars, and wrinkles, such as in the neck and chest, buttocks, abdomen, arms, thighs, knees, and hands. For patients with nasal implant surgery scars and contractures, other fillers pose a high risk due to unpredictable vessel orientation. However, PLLA has almost no risk of embolism, and using this material for contouring in scar areas can significantly reduce the risk of embolism [14][15][16].

After rhinoplasty, some patients may experience thicker capsules and subcutaneous scars, which not only severely affect the skin’s elasticity but also cause deeper scar infiltration due to centripetal contraction caused by infection. It may also lead to mucosal contraction, folding, and worsen the difficulty of repair. Therefore, treating contracted nose is one of the most difficult surgeries in rhinoplasty [17]. For patients with nasal scar contracture, the skin and soft tissue are most affected. During the scar softening process, we first guide the patient to traction the skin and soft tissue by themselves, which can improve skin elasticity and reduce the difficulty of surgery before the operation [18][19]. During the surgery, pay attention to the appropriate removal or cutting of the silicone capsule, and judge where the resistance that affects skin elasticity and extension comes from when using the double-toothed hook for downward traction. Gradually release the scar and remove the capsule to obtain sufficient skin and soft tissue [20]. It is necessary to use a progressive method to cut the scar contracture band to fully release it, but at the same time, observe the blood supply of the nasal tip to avoid damage and serious complications such as skin necrosis. Since the nose involves multiple subunits and is a three-dimensional structure, in addition to having good filling and repair materials, surgical techniques and comprehensive surgical plans are also one of the main influencing factors for the postoperative repair effect of nasal dorsal scars [21].

## Conclusion

Inject able PLLA demonstrates promising efficacy and safety for the treatment of depressed acne scars, particularly for boxcar and rolling scars. The treatment also provides overall improvements in skin quality, including enhanced luster, smoothness, and pore appearance. While improvements in icepick scars were more modest, the multi-faceted benefits of PLLA suggest its potential as a valuable addition to the treatment armamentarium for acne scarring.

The high patient satisfaction rates and favorable safety profile observed in theresearch further support the clinical utility of PLLA in this context. However, the differential response among scar types highlights the importance of careful patient selection and setting appropriate expectations, Based on the above, in the treatment of scar release, PLLA has a good effect on repairing the scar contracture on the patient’s nasal dorsum and is worthy of further research. However, this study is only a case report, which may lead to biased results. In addition, due to differences in patient physiological conditions, etc., a large sample, multi-center, high-quality evidence-based medical research is still needed to further support these findings.

## Conflict of Interest

None.
